# Re-analysis of data from cluster randomised trials to explore the impact of model choice on estimates of odds ratios: study protocol

**DOI:** 10.1186/s13063-024-08653-1

**Published:** 2024-12-18

**Authors:** Karla Hemming, Jacqueline Y. Thompson, Monica Taljaard, Samuel I. Watson, Jessica Kasza, Jennifer A. Thompson, Brennan C. Kahan, Andrew J. Copas

**Affiliations:** 1 Institute of Applied Health Research, University of Birmingham, Birmingham, UK; 2 Clinical Epidemiology Program, Ottawa Hospital Research Institute, Ottawa, Canada; 3 School of Epidemiology, Public Health and Preventive Medicine, University of Ottawa, Ottawa, Canada; 4 School of Public Health and Preventive Medicine, Monash University, Melbourne, VIC Australia; 5 Department of Infectious Disease, London School of Hygiene & Tropical Medicine, London, UK; 6 MRC Clinical Trials Unit at UCL, University College London, London, UK

## Abstract

**Background:**

There are numerous approaches available to analyse data from cluster randomised trials. These include cluster-level summary methods and individual-level methods accounting for clustering, such as generalised estimating equations and generalised linear mixed models. There has been much methodological work showing that estimates of treatment effects can vary depending on the choice of approach, particularly when estimating odds ratios, essentially because the different approaches target different estimands.

**Methods:**

In this manuscript, we describe the protocol for a planned re-analysis of data from a large number of cluster randomised trials. Our main objective is to examine empirically whether and how odds ratios estimated using different approaches (for both primary and secondary binary outcomes) vary in cluster randomised trials. We describe the methods that will be used to identify the datasets for inclusion and how they will be analysed and reported.

**Discussion:**

There have been a number of small comparisons of empirical differences between the different approaches to analysis for CRTs. The systematic approach outlined in this protocol will allow a much deeper understanding of when there are important choices around the model approach and in which settings. This will be of importance given the heightened awareness of the importance of estimands and the specification of statistical analysis plans.

**Supplementary Information:**

The online version contains supplementary material available at 10.1186/s13063-024-08653-1.

## Background

Cluster randomised trials (CRTs) are characterised by the non-independence of observations within clusters; this intra-cluster correlation must be accounted for in the analysis to obtain valid inferences. There are several ways to allow for the non-independence of observations at the analysis stage of a CRT. When analysing individual-level data, the two main approaches are generalised linear mixed models (GLMM) and generalised estimating equations (GEE) [1–5]. When the treatment effect is estimated using odds ratios, these two approaches yield different interpretations. In particular, (i) the GLMM yields a cluster-specific estimate, i.e. the effect of the treatment conditional on cluster membership, and (ii) the GEE yields an unconditional (marginal) estimate. Thus, it is often said that in the analysis of cluster trials, when using individual-level data with binary outcomes, and when reporting odds ratios, the choice of approach (between GEE and GLMM) should be guided by whether the researcher is interested in the marginal or conditional effect of the treatment [6]. This is important because the odds ratio is one of the most commonly reported summary measures of treatment effects for binary outcomes in CRTs [2].

Yet, there is also another approach to the analysis, using what is known as a cluster-level analysis [7]. Essentially, these proceed by aggregating cluster-level outcomes using a summary statistic (such as the cluster proportion in the case of a binary outcome) and then applying a conventional analysis method to these summary statistics (for example, a *t*-test of the cluster proportions). If the cluster proportions are logit-transformed prior to analysis, the application of the *t*-test yields estimated odds ratios. These cluster-level analyses can be unweighted (so that every cluster makes the same contribution to the analysis regardless of its size), size-weighted (so larger clusters make a larger contribution to the overall treatment effect, not dissimilar to weighting in a meta-analysis by study size), or weighted using minimum variance weights (again, not dissimilar to a meta-analysis using inverse variance weights; this approach is taken less frequently) [8]. The choice between an unweighted or size-weighted approach often depends on desired statistical efficiency but also depends on whether the target of inference is (i) the effect of the intervention on a typical individual (targeted by a size-weighted analysis) or (ii) the effect of the intervention on a typical cluster (targeted by an unweighted analysis). In settings where cluster sizes are informative—which essentially means either the outcomes depend on cluster size and/or the treatment effect depends on cluster size, these different approaches to a cluster-level analysis can realise different estimates of odds ratios (and indeed differences between other summary measures, such as the relative risk)—again because they are targeting different treatment effects [9].

Recently, it has been suggested that in settings where there are informative cluster sizes, neither the GLMM nor the GEE (with an exchangeable working correlation structure) targets the effect of the treatment on the typical individual. Rather, when using individual-level data and when the target of inference is the effect on the typical individual, an approach based on independence estimating equations (IEE) (which applies GEEs with an independent working correlation structure) should be used [9–11]. The IEE approach is equivalent to what is called a cluster-robust approach [9, 12] and is used much less frequently in practice [3, 4]—in part because it has larger standard errors, wider confidence intervals and lower statistical precision and thus results in lower statistical power [11]. In practice, within any given setting, it can be extremely difficult to discern whether cluster sizes are informative; testing for interactions between cluster size and treatment, for example, is likely to suffer from the same sorts of issues that arise when trying to detect any sub-group effect; and comparing effects from models that target the average cluster effects to those that target the average individual effect can also be challenging. Moreover, considering the types of complex interventions evaluated in many CRTs, intervention effects might well vary by cluster size.

Thus, in situations where it is possible that the intervention effect differs for clusters of different sizes, researchers must consider whether they are interested in the individual or cluster-level average. Researchers must also choose if they are interested in the marginal or conditional effect. Eliciting what are the targets of inference can be a challenge: statisticians might have difficulty in understanding these subtle differences; they are then tasked with communicating these subtleties to clinical research partners—the meaning of which might get lost in translation—and/or clinical partners might have difficulty in understanding these differences.

Therefore, we propose to evaluate, over a large sample of datasets on primary and secondary binary outcomes from CRTs, whether there is evidence of any material difference in point estimates and standard errors when estimating odds ratios from a range of potential analysis approaches. Whilst such an empirical evaluation cannot settle the debate about whether targets of inference are more appropriately aligned with the marginal or conditional effect nor whether there is or is not evidence of informative cluster size, it might be able to shed light on how much of a difference the different approaches can have across a large number of non-selective datasets and particularly allow examination under which circumstances the approaches are more likely to differ empirically.

## Aims and objectives

The overall aims for the work outlined in this protocol are to obtain a large number of datasets from completed CRTs, re-analyse these datasets using a series of common analysis approaches and empirically explore whether and how estimated odds ratios vary across different model choices.

## Methods

We outline the inclusion and exclusion criteria, the approaches to obtaining datasets and how the datasets will be stored and analysed and outline the candidate analysis approaches with justification, including anticipated relationships.

### Inclusion and exclusion criteria

Our objective is to compare analysis methods for a large number of CRTs. We make a number of pragmatic decisions around inclusion criteria and so limit our comparisons to more standard parallel designs with at least 10 clusters (for cluster trials with fewer than 10 clusters, model-based standard errors are at a much greater risk of being biased, even with small sample corrections [13]). The criteria for a study to be included in this re-analysis project are:

1. Cluster randomised parallel arm trial (or parallel with baseline period)
2. Two or more arms
3. Not pilot or feasibility
4. Superiority or non-inferiority
5. At least 10 clusters in total
6. Published report in a peer-reviewed journal
7. At least one binary outcome available (not necessarily primary)

In the comparative analysis of different approaches, we will include only two arms from any identified multiple-arm trials (see later details for how we will choose two arms where there are more than two arms), and whilst we will collect both binary and continuous outcomes, in this initial analysis plan we will only consider binary outcomes. We include trials in which clusters are randomised to arms in which a baseline period is included (either cohort or cross-sectional), but in the analysis, we only include the post-randomisation observations. We will exclude cluster-randomised designs in which clusters switch between interventions (e.g. stepped-wedge cluster randomised trials). There are no date requirements other than those listed specifically below, but all studies must have been published in the English language (so as to facilitate understanding of the trial design, which will be important in the steps to extract the appropriate data, below).

### Methods to identify and obtain datasets

We will use three different methods for identifying and obtaining datasets. Each of these are outlined below.

1. Datasets identified via a large review of CRTs.

An existing database of nearly 800 CRTs in lower-middle-income countries (LMICs) was compiled for a review of CRTs with a different objective and reported elsewhere [14]. We plan to use this database out of convenience and because it is a resource for a large number of CRTs. The search for the original review was implemented in Ovid MEDLINE on August 17, 2022; it was limited to articles published in the English language between January 1, 2017, and the search date. On April 18 2024, using Web of Science, we retrieved 780/798 records from the 798 PubMed IDs that were already available from the 800 CRTs identified in the review; amongst those 780 records, 777 had email addresses (we will obtain the remaining 22 email addresses by manually identifying the corresponding author from the trial). We will include the subset of those studies with identified email addresses and that also meet the criteria listed above (e.g. of the 800 CRTs, 713 of these have a parallel design). We will contact the authors of each of the studies via the email address available. We will share a copy of our study protocol and ethical approval documentation. A copy of this initial email, which outlines our objectives, and the specific requests of the study team is included in the supplementary material (Supplementary Material 1). In cases of non-response, we will attempt two more follow-up attempts approximately 2 weeks apart (taking care to avoid known holidays). In the case of further non-response, we will identify another key author from the paper and attempt to make a final contact. An ongoing project similarly attempted to obtain datasets from a systematic review of 160 stepped-wedge cluster trials, resulting in the sharing of data from approximately 20%. We, therefore, anticipate obtaining access to approximately 143 datasets.

2. Datasets known to the authors.

The authors all work in CRTs and have collectively analysed a large number of parallel cluster trials. All authors will each ask for permission from principal investigators of CRTs they have worked on to share the datasets known to them. The method of contact will be as described above for the LMIC sample.

#### Datasets available as publicly shared datasets held on data-sharing sites

There are a number of websites that host medical datasets that are publicly available. We will search these datasets for CRTs. Specific search strategies will be developed for each website, after careful scoping work to ensure that the databases are likely to contain appropriate detail. Relevant websites known to the authors are listed in Supplementary Table 1.

### Data collection items

We will retain a record of all invitations sent, responses received and a record of all the datasets received (including basic information such as a link to the trial registration, the PDF of the final trial report and the contact details of the investigator who shared the data). We are primarily requesting individual-level data as a fundamental part of our objectives, which is to estimate effects using an individual-level analysis. However, cluster-level data (i.e. numerator/denominator data) could also be analysed at the individual-level (see analysis section for details), and this may be a way to obtain sensitive data and avoid investigators having to share individual-level records. We will also provide the option that data are not shared with us, but rather, a local study statistician runs the code we provide. For trials where the data are already publicly available, the available data will be downloaded directly. We will check for duplicates between publicly shared datasets held on data-sharing sites and records identified via the large review of CRTs. We will not contact authors of CRT whose data we already possess. For trials where it is necessary to approach investigators to share data, we will ask for the following data from each identified trial:

Individual-level datasets:

1. Cluster indicator
2. Treatment arm indicator
3. Any key primary or secondary binary outcome variables
4. Any key primary or secondary continuous outcome variables
5. Any cluster-level variables used in any restricted randomisation
6. Any individual or cluster-level variables used in a covariate-adjusted analysis

Cluster-level datasets:

1. Cluster indicator
2. Treatment arm indicator
3. Any key primary or secondary outcome variables:
  - For binary outcomes—count of the number of occurrences of the outcome in the cluster (along with the number in each cluster contributing to that outcome (i.e. cluster size))
  - For continuous outcomes—cluster-level means (and cluster size)
4. Any cluster-level variables used in any restricted randomisation

#### Datasets not available for sharing

Where an investigator indicates that they are unable to share a dataset but agrees to the analysis being undertaken by the local study statistician, we will share the code for the analysis (in Stata only)—and instructions for implementation. The study statistician would then return estimates of log-odds ratios and standard errors for all of the candidate approaches (along with descriptive characteristics of the study, such as the number of clusters—see later details). Replicating models between different software packages can be challenging, and to avoid this complexity, we will not offer this facility in other packages.

#### Justification for data collection items

We plan to ask for secondary and primary outcome variables to maximise the number of outcomes we have for our analysis, which will increase the likelihood that we are able to make informative conclusions. We will limit our request to key primary and secondary outcomes and so include only pre-specified secondary outcomes in the trial registration or protocol (and so, for example, will exclude safety outcomes). Including multiple outcomes from each study will greatly enhance the number of outcomes available for analysis and will increase the generalisability of the findings. This proposal focuses on estimating odds ratios from binary outcomes to limit the scope. Future work could look at other outcome types (e.g. continuous outcomes) or other summary measures (e.g. risk difference), and for that reason, we will also collect continuous outcomes if available. We also will collect information on any key covariates available (those used in the randomisation and any used in an adjusted analysis) as covariate adjustment can be a very useful way to increase study power and can, in some settings, change estimated effects (this is particularly possible in CRTs with post randomisation identification or recruitment biases [15])—and demonstrating this could also be empirically useful. However, again, whilst we capitalise on this opportunity to collect this additional data, this proposal does not consider the use of covariates explicitly.

### Data storage and protection

#### Data storage

Data will be requested at the individual level but in a de-identified form so that it is fully anonymised, and we will not have any information about individual participants. The University of Birmingham, Edgbaston, Birmingham, B15 2TT, is the data controller. All datasets will be stored on password-protected University of Birmingham secure servers or password-protected university computers; and the only people who have access to these datasets will be those who are performing the statistical analysis. An anonymised version of each dataset (which will not contain an identifiable link to the primary studies) will be created and used as the working version of the dataset (this will not be password protected) and will be retained for 10 years after the publication of the research outcomes for this initial proposal. This anonymous version of the data will be stored on the University eData repository (https://edata.bham.ac.uk).

#### Data sharing

We will not share the datasets with any third party. This protocol contains specific details for the project, which aims to determine if different approaches to analysis have an impact on inference for the odds ratio. The datasets may also be used (only by the study team) to answer related questions, including whether the approaches to analysis have an impact when estimating (i) risk ratios, (ii) mean differences for continuous outcomes, (iii) covariate-adjusted effects and (iv) comparisons across statistical packages, for example. This will only be undertaken by members of the study team and will be subject to further ethical approval.

#### Identification of trials

The objectives are to identify, across a large sample of trials, if there are differences between the different approaches. Our objectives do not relate to identifying whether particular treatments work or do not work. For this reason, we will not identify the trials or the treatments by the names or authors in any reported results.

### Pilot phase

We will pilot all aspects of our approach. For example, we will pilot-test the email approach. To this end, we will send out the email requests in an initial batch of ten. This will allow for the refinement of the invitation and modification of issues that might be unclear. Furthermore, once we have received ten datasets, we will carefully test the approach for storing data and analysis code to run the models. We anticipate only reaching out to ten studies each time, so as to ensure we do not become overloaded with responses and that we have time to individualise our responses to maximise the usefulness of the data we receive.

### Data analysis

#### Statistical analysis objectives

Our overarching objective is to identify if, amongst all of the candidate analysis approaches, there are differences that would make a material impact on the inference of the treatment effect. This overarching objective is broken down into three broad objectives to determine:

i. To explore and characterise differences (in point estimates, standard errors, confidence intervals) between estimates obtained from the current dominant approaches (e.g. a mixed model compared to generalised estimating equations using an exchangeable correlation structure)
ii. Whether there are differences between the different estimands (e.g. are marginal estimands typically different to cluster-specific estimands)
iii. Whether, for the same estimand, there are differences between different estimators (e.g. for a marginal participant-average estimand, is there a difference between using independent estimating equations and generalised estimating equations assuming an exchangeable working correlation structure?)

For each of these broad aims, we propose a number of different analysis approaches (Table 1) and have a number of specific aims (Table 2). The motivation for consideration, details of any anticipated relationships and approach to be taken to investigate the relationships are all detailed in Table 2.

**Table 1:** Summary of the proposed analysis approaches

Label	Model	Standard error	Estimand: marginal or cluster-specific	Estimand: cluster-level or participant-level average	Comments	Stata code
Naïve	GLM with logit link	Model-based asymptotic (observed information matrix)	Marginal	Participant-average	No allowance for clustering	glm outcome treat, family(binomial) link(logit) iterate (1000) Maximum likelihood using Newton–Raphson
GLMM	GLMM with logit link	Model-based asymptotic (observed information matrix)	Cluster-specific	Unclear^a^	With cluster robust in limited settings (See Aim S1)	meglm outcome treat || cluster:, family(binomial) link(logit) iterate(1000) Integration method: mean–variance adaptive Gauss-Hermite quadrature; Maximisation method: Newton–Raphson Robust version meglm outcome treat || cluster:, family(binomial) link(logit) iterate(1000) vce(cluster cluster)
GEE	GEE with logit link, exchangeable working correlation	Huber/White/sandwich estimator (Robust)	Marginal	Unclear^a^		xtgee outcome treat, family(binomial) link(logit) corr(exchangeable) vce(robust) iterate(1000)
Marginal GLMM	GLMM with logit link and marginalised over clusters	Huber/White/sandwich estimator (Robust)	Marginal	Unclear^a^	The variance–covariance matrix is estimated (VCE) is estimated using the linearisation method	meglm outcome i.treat || cluster:, family(binomial) link(logit) iterate(1000) vce(robust) margins treat, post vce(unconditional) nlcom (_b[1.treat]/( 1-_b[1.treat]))/(_b[0.treat]/(1-_b[0.treat])), post
IEE	IEE with logit link	Huber/White/sandwich estimator (Robust)	Marginal	Participant-average		xtgee outcome treat, family(binomial) link(logit) corr(independent) robust iterate(1000)
IEE size-weighted	IEE with logit link and size weights	Huber/White/sandwich estimator (Robust)	Marginal	Cluster-average		xtgee outcome treat [pw = 1/cluster_size], family(binomial) link(logit) corr(independent) robust iterate(1000)
CL	LM fitted to logit of cluster-level proportions	Model-based asymptotic (observed information matrix)	Cluster-specific	Cluster-average		glm logit_prop treat iterate(1000)
CL size-weighted	LM fitted to logit of cluster-level proportions weighted by cluster size	Huber/White/sandwich estimator (Robust)	Cluster-specific	Participant-average	Robust SE to protect against heteroskedasticity when cluster sizes vary	glm logit_prop treat [pweight = cluster_size], family(gaussian) link(identity) iterate(1000) ^a^robust standard errors provided by default in Stata
CL-GLM	GLM fitted to cluster-level proportions with logit link [reference]	Model-based asymptotic (observed information matrix)	Marginal	Cluster-average		glm prop treat, family(normal) link(logit) iterate(1000)
CL-GLM size-weighted	GLM fitted to cluster-level proportions with logit link with size weights [reference]	Huber/White/sandwich estimator (Robust)	Marginal	Participant-average	Robust SE to protect against heteroskedasticity when cluster sizes vary	glm prop treat [pweight = cluster_size], family(normal) link(logit) robust iterate(1000)

Stata code: prop is proportion per cluster; logit_prop = the log odds in the cluster; treat is a binary treatment indicator; cluster is a variable indicating cluster; outcome is a binary outcome indicator for each individual; outcome_total is the total count of positive outcomes in cluster; cluster_total is the count of total number of observations in cluster

*GEE* Generalised estimating equations, *LM *Linear model, *GLM *Generalised linear model, *GLMM *Generalised linear mixed model. Note in Stata, although when fitting LM with robust SEs these are what are known as HC1 standard errors, for GLMM, it is unclear if the Stata robust SEs are HC1 or something different

^a^Unclear regarding the participant vs. cluster-average aspect for mixed-models and GEEs with an exchangeable correlation structure. This is because these methods do not target a clear estimand except under the assumption of no informative cluster size (in which case they target both the participant- and cluster-average effect, which should be the same)

**Table 2:** Aims and motivation for proposed analysis with the intended presentation of findings

Aim	Aim and motivation	Compared approaches	Figure number	Key variables for consideration
***Comparison of standard errors***				
S1	Confirm that approaches that do not allow for clustering have smaller SEs than other approaches. Anticipate the approach which does not allow for clustering will have smaller SEs than other approaches, with the exception of settings with a very small ICC where the SE from the naïve approach will be similar [28].	SE from GLM vs. four other SEs (one for each of the four estimands)	S1	ICC
S2	Determine if models with cluster-robust standard errors typically have larger standard errors than the other approaches. Anticipate that the GLMM approach with cluster-robust SEs will have a similar or larger SE compared to either the GLMM, GEE or IEE approach without robust SEs [29].	SE from GLMM robust vs. four other SEs (one for each of the four estimands)	S2	ICC
***Objectives motivated by comparison of dominant approaches***				
1	Determine how the current dominant approaches to analysis (i.e. GEE with an exchangeable correlation structure and GLMMs) compare to each other. Anticipate there are likely to be minimal differences between cluster-specific vs. marginal approaches when the ICC is close to 0; the SEs from the GLMM to be typically larger than those from GEE; the point estimate from the GEE is typically smaller than the point estimate from the GLMM (or there is a minimal difference) for larger ICCs [17].	GLMM vs. GEE	1	ICC
2	Determine how the current dominant approach to analysis, when only cluster-level data are available, compare (CL size-weighted approach vs CL unweighted approach). Anticipate that there will be large differences when both cluster sizes vary and there are differences in outcomes, or the effect varies across clusters [7].	CL size-weighted vs. CL unweighted	2	CV of cluster sizes; correlation between prevalence and size
3	Determine how point estimates from the GEE with an exchangeable correlation structure (the dominant approach to estimate the marginal effect) and those from an IEE unweighted approach (a hypothesised alternative to the estimation of the marginal effect in settings of informative cluster size) compare to each other. Anticipate there are likely to be more differences when cluster size varies; there are differences in outcomes/effects across clusters of different sizes; and a non-negligible ICC [11].	IEE vs. GEE	3	ICC CV of cluster sizes; Correlation between prevalence and size
***Objectives motivated by comparisons across estimands***				
4	Determine whether the ***marginal effect*** (as derived from independence estimating equations) is similar to ***the cluster-specific effect*** (as derived from a cluster-level analysis weighted by cluster size). Both approaches target participant-level averages (Table 1, second estimand column), but IEE targets the ***marginal effect,*** and the CL size-weighted approach targets the cluster-specific effect. Anticipate there to be differences between IEE and the CL size-weighted and this would indicate that due consideration is required around whether the estimand is the ***marginal effect*** or cluster-specific effect (marginal vs cluster-specific) [9].	CL size-weighted vs IEE	4	ICC
5	Determine whether the ***participant-average effects*** (as derived from independence estimating equations) and the ***cluster-average*** (as derived from size-weighted independent estimating equations) differ in practice. Both approaches target marginal effects (Table 1, first estimand column), but IEE targets the participant-average, and the size-weighted IEE analysis targets the cluster-average effect. Differences would indicate that due consideration is required around whether the estimand is the participant-average or cluster-average effect (for marginal effects). Note aim 2 above will identify if the participant-average and cluster-average effects differ for conditional effects [11].	IEE size weighted vs. IEE	5	ICC CV of cluster sizes; correlation between prevalence and size
S3	Determine if the expected theoretical relationship between the population-averaged effect (i.e. the point estimate from GEE with an exchangeable correlation structure) and the cluster-specific effect (i.e. the point estimate from the GLMM) holds in practice. A formula for the theoretical and approximate relationship between the point estimate from the GEE and the point estimate from the GLMM is available [17]. The theoretical relationship depends on the variance of the random effect, with the population average effect being less than or equal to the cluster-specific effect [17]. This theoretical formula makes a number of assumptions (for example, that there is no ICS), and others have already shown across a limited number of example datasets that this relationship does not always hold in practice [18].	Theoretical relationship vs observed ratio of GLMM/GEE (on OR scale, not log OR scale)	S3	ICC
**Objectives related to comparisons of approaches that target the same estimand**				
S4	Comparison of approaches that target the marginal cluster-level average [30].	IEE size-weighted vs. CL linear logit	S4	
NA	Comparison of approaches that target the conditional cluster-level average. There is only one included approach that targets this—so no comparison here is needed.	CL unweighted;		
S5	Comparison of approaches that target the marginal participant-level average.	Naïve vs. IEE vs. CL linear logit size-weighted	S5	
NA	Comparison of approaches that target the conditional participant-level average. There is only one included approach that targets this—so no comparison here is needed.	CL size-weighted		
S6	Comparison of approaches that target the marginal effect in the absence of informative cluster sizes.	GEE vs marginalised GLMM	S6	

For definitions of approaches, see Table 1, see supplementary figures

#### Standardisation of datasets

We will standardise datasets for analysis. The unit of randomisation will be defined as ‘cluster’; the binary outcomes coded as outcome_1 (primary if available) to outcome_X; the intervention indicator coded as ‘treatment’ (1 treated; 0 control). In cases of more than two arms, we will include the control arm (standard of care) and only one of the other treatment arms (we will retain the arm with the largest number of clusters or observations). In cases of more than one time period, we will include the first (post-randomisation) assessment time period. We will remove all pre-randomisation baseline assessment periods. We will exclude any studies that have fewer than 10 clusters available for analysis. This will thus result in a dataset for each study, which has a number of binary outcomes, two treatment arms, more than 10 clusters and a single follow-up assessment time. Each outcome from each trial will be analysed separately. For datasets provided in a cluster-level form (cluster-level data on: indicator, numerator and denominator), these will be converted into individual-level data by reformatting so there is one row in the dataset to represent each observation per cluster (e.g. each cluster will be expanded to include number of rows equivalent to the denominator with the outcome coded as one for the numerator for that cluster).

Two of the approaches (the standard cluster-level analysis and the size-weighted cluster-level approach, Table 1) both exclude clusters with zero events by default. To compare the performance of these two approaches across trials, including those with zero events in any clusters, we, therefore, adapt this method by adding half an event to all clusters with zero events [7, 8, 15, 16]. This correction will be made to each outcome dataset before any analyses, and this corrected dataset will be used for all analyses to allow a fairer comparison.

For each dataset and each outcome, we will also compute the cluster size to implement the size-weighted analysis. To this end, for each dataset and for each outcome, we will sum the number of observations that contribute to that outcome for that dataset. When we implement the size-weighted analyses, we will use the size of the cluster with respect to the outcome in question (and so, for example, we would not count participants with missing outcome data).

#### Descriptive summary of studies included

We will first report a descriptive summary of the studies included (a dummy version of this Table is included in Supplementary Table 2). This will include the number of unique studies and outcomes available, and the average number of outcomes available for each study. We will then report a number of summary measures that describe the outcomes available. Summary measures to be reported include the total sample size, the average cluster size; the number of clusters and the coefficient of variation of cluster sizes across clusters; the proportion with the outcome in the control arm and the coefficient of variation of this proportion across clusters; the intra-cluster correlation coefficient (reported on the proportions scale, estimated using a linear mixed model, and adjusting for treatment [17]); and the correlation between cluster size and cluster outcome proportion within the control arm. These characteristics will all be summarised using medians and interquartile ranges and relate to the numbers available for each outcome (e.g. the sample size relates to the sample size available for that outcome).

#### Candidate model approaches

Data will be analysed using a number of different approaches to analysing cluster data (Table 1). These approaches have been chosen to cover the combination of marginal, conditional, cluster-level and individual-level targets. These approaches also include approaches that are commonly used and accepted as standard. The candidate model approaches are all informed and justified on the basis of the statistical analysis objectives. In Supplementary Table 3, we include methods that were not considered, with a justification for exclusion.

#### Candidate factors which might influence the relationships

When exploring whether there are differences between the different approaches, we consider the following factors which are known to be of importance:

- Number of clusters (known to be of importance for estimating standard errors without bias) [13]
- Event rate (known to be of importance in the difference between marginal and conditional effects) [17, 18].
- Intra-cluster correlation coefficient (ICC) on the proportion scale (known to be of importance in the differences between GEE and GLMM approaches [18, 19]
- Coefficient of variation (CV) of cluster sizes (known to be of importance when there are informative cluster sizes [11])
- Correlation between cluster size and cluster outcome proportion in the control arm (a simple measure of one type of informative cluster size) [11]
- Point estimate (bias might be greater when the effect size is further away from zero) [8]

For the most part, the impact of these factors will be investigated by retaining each factor in its continuous format (e.g. by depicting one factor in its continuous format on the *x*-axis of a figure—see Supplementary Fig. 1 for example where the coefficient of the cluster sizes is presented on the *x*-axis). We will, however, also categorise into sub-groups to allow multiple factors to be displayed on figures (e.g. ICC as depicted in Supplementary Fig. 1). The precise cut-points for these categorisations will be determined using a combination of data availability and anticipated important thresholds and, for example, would consist of: ICC ≤ 0.001; 0.001 to 0.05; ≥ 0.05; CV of cluster sizes ≤ 0.25; 0.25 to 0.75; ≥ 0.75. Finally, the analyses will be run across all datasets and limited to datasets with more than 40 clusters, where we can be more confident that standard errors will be unbiased.

#### Analysis implementation

We will use Stata 18 to implement all model fitting, and details of the code is provided in Table 1. To fit GLMs, we use the *glm* command in Stata. This is contrary to Stata documentation, which recommends that where there is an overlap between the capability of *glm* and another command, the other command should be used (e.g. use *logit* instead of *glm*). However, the use of *glm* and *meglm* produces a more standardised output that is slightly easier to work with. We limit the number of iterations to 1000 for computational feasibility. We use robust standard errors where appropriate with justification and maximum likelihood estimation using Newton–Raphson (default in Stata) and mean–variance adaptive Gauss-Hermite quadrature (default in Stata), and we mostly follow the default options in Stata. 95% confidence intervals will be created using t-distribution with the number of clusters minus two degrees of freedom.

We will present findings on the log-odds scale and the standard error of the log-odds ratio. In an individually randomised trial, conditional estimates tend to be larger than marginal ones and have correspondingly larger standard errors [20]. This is likely to be similar in CRTs. Thus, an estimate that targets a conditional/cluster-specific effect will have a larger estimate than one which targets a marginal effect and should also have a correspondingly larger standard error. Our objectives here are to simply consider this as a hypothesis and to determine if, in practice, it is the case that standard errors for conditional effects are typically larger than those of marginal effects. For this reason, we do not consider different scales—such as statistical power.

For each comparison of any two candidate approaches, we will report in graphical format (i) a comparison of the log-odds ratio from each of the two candidate approaches (e.g. point estimate from GEE vs. point estimate from IEE), (ii) the ratio of the two-point estimates of the log-odds ratio (e.g. point estimate from GEE/point estimate from IEE vs. point estimate from IEE) and differences on a percentage scale (e.g. point estimates from (IEE-GEE/IEE)*100 vs point estimate from IEE, (iii) a comparison of the standard errors from the two candidate approaches (e.g. the standard error from the GEE approach vs. standard error from the IEE approach) and (iv) a comparison of the confidence intervals from the two candidate approaches (e.g. the confidence interval from the GEE approach vs. confidence interval from the IEE approach). A detailed list of all of the two-way comparisons of candidate approaches is detailed in Table 2. We will explore the use of presenting on a log scale and truncating the axis for outliers.

#### Planned interpretation of findings

Our interpretation will focus on when results are mostly similar (i.e. confidence intervals are mostly overlapping). For example, we expect to show that the point estimates from the marginal and conditional approaches show a strong one-to-one correlation. We will also highlight when there may be differences. For example, we expect to show that there are proportionately larger differences when the point estimate is close to zero. Case studies will be selected to illustrate these points. For example, a case study for which there is a clear effect (and so the point estimate is not close to zero) and comparing the marginal and conditional approaches should illustrate that on the odds ratio scale both approaches suggest large effects (and so differences between effects largely inconsequential), whereas a case study for which there is evidence of no, or a very small, effect (and so the odds ratio close to zero) should illustrate that both approaches can lead to different effect estimates, which might be of importance if small effects are clinically important. Factoring into this interpretation, confidence intervals and the range of uncertainty will also be considered. We therefore aim to provide recommendations for when the dominant approaches can be used without having to consider the sensitivity of findings to alternative approaches as well as establishing factors which might be used as signals that results might be sensitive to approach.

### Feedback on study results to participants

We will share results with all study investigators who provided datasets. Every study investigator will be asked if they wish their study to be acknowledged (by the names of the people who facilitated the sharing of the data, the study name, and publication).

## Discussion

There have been a number of small comparisons of empirical differences between the different approaches to analysis for CRTs. For example, an analysis of four datasets using a limited number of approaches identified limited variation across approaches [21]; whereas an analysis of a single CRT, but with only a small number of clusters, called the Trigger trial, revealed seemingly large differences between different approaches [11, 22]. The systematic approach outlined here for the identification and analysis of datasets from cluster randomised trials will allow a much deeper understanding of when there are important choices around the model approach, and in which settings, and this will be of importance given the heightened awareness of the importance of estimands [23] and the specification of statistical analysis plans [24, 25].

### Limitations

#### Generalisability of findings

Our findings will be limited by both the number of datasets we are able to obtain and their representativeness. Increasing the number of datasets will reduce the possibility of our findings being influenced by outliers but will be limited by the number of responses to our requests for data sharing. The generalisability of our findings might be limited by how one of our primary sources for datasets is a review of CRTs limited to LMICs. However, there is no known reason why this should impact our study question. Furthermore, we will present some of our findings by key factors that are known to be influential, such as the number of clusters, the intra-cluster correlation coefficient and event rate.

#### The importance of research input into the estimand

We underscore the importance of how model choice should be dictated by the target estimand and not the other way around. However, even in the individually randomised trial literature, there is considerable debate about when marginal and conditional effects should be the target of inference [26–28]. We, therefore, hope that this empirical evaluation might complement other ongoing research into this very important aspect of trial design and analysis.

#### What is an important difference

The interpretation of what constitutes an important difference between approaches will be nuanced. Furthermore, even if we identify that differences are only important under various scenarios (e.g. high ICCs), because the factors that drive the differences will not be known at the design stage, it would be difficult to conclude that these constructs do not need careful consideration for a given trial, as it will be hard to rule out differences entirely at the design stage.

#### Small sample corrections

Many of the trials included will include only a small number of clusters, and so the standard errors will be biased downwards. Making small sample corrections to these standard errors, however, is not straightforward. We have opted for a pragmatic approach to small sample corrections (e.g. by using the between-within correction for mixed models and cluster-level analyses and robust standard errors for marginal approaches). These might not be optimal in all settings (for example, in some specific scenarios, one small sample correction might perform better than another [13, 29]). Yet, our focus is primarily on the point estimates. Simply describing the pattern of standard errors across the datasets and across the analysis approaches is a secondary objective for completeness only.

#### Informative cluster sizes

We will not be able to differentiate or determine what part of any differences have to do with informative cluster sizes—for this, more theoretical or simulation-based studies are required. In addition, informative cluster sizes might relate either to the effectiveness of the intervention as a function of the actual/real-world cluster size or the effectiveness of the intervention as a function of the observed/sampled cluster size. However, we will be able to investigate whether differences between approaches are more prevalent when cluster sizes vary or by the correlation between cluster size and cluster outcome proportion.

#### Covariate adjustment

One of our key objectives is to determine how much the cluster-conditional and cluster-marginal estimands differ. There may, however, also be potential differences between the effects, conditional and unconditional on covariates [20]. A future project may explore the additional complexities of including covariate adjustment (including baseline values of the outcome). However, there will be difficulties with operationalizing the choice of covariates for adjustment (e.g. looking at statistical analysis plans to inform which covariates to adjust for could be time-consuming and likely under-reported).

#### Different outcomes or treatment effect metrics

We only consider binary outcomes and limit consideration only to odds ratios. Other treatment effect measures include relative risks, risk differences and mean differences, and these can also be impacted by informative cluster sizes, whereas only hazard ratios and odds ratios target different marginal and conditional effects. Again, a future project may explore how relationships hold for different outcome types and different summary measures of effects (e.g. risk difference).

#### Comparison of power/statistical precision

We aim to compare standard errors across the different approaches. An alternative would be to understand whether certain methods are leading to an increase in statistical power. In which case, reporting log(OR)/SE(log(OR)) would be an alternative. However, (i) it is not clear exactly how this maps onto statistical power, and (ii) this would assume that the estimated log(OR) from any given candidate approach is unbiased, and if it is not, then this measure is no longer useful. Thus, whilst this works well for unbiased estimators, for biased estimators (such as generalised estimating equations or mixed-models in the presence of informative cluster sizes), this provides a combined measure of bias/precision, which is difficult to interpret. Thus, a larger log(OR)/SE value could either denote increased precision/power, or increased bias. Thus, trying to demonstrate any increase in statistical power/precision from empirical data is challenging, and so we thus limit our consideration to standard errors only. We also assume no systematic error in the estimated standard errors and will not investigate whether any differences seen in precision between estimators are due to bias.

#### Clusters with zero events

There are indeed various approaches to accommodating zero events in a cluster-level analysis. For example, adding 0.5 to the numerator and 1 to the denominator [8] or 0.5 to the numerator [7] or 0.5 to both the numerator and the denominator [30]. Furthermore, in studies with small cluster sizes (where zero events in clusters are more typically likely), the cluster-level analysis has been observed to provide biased estimates, which might suggest that these approaches are not optimal [8].

#### Alternative approaches not considered

There are a number of other potential approaches we have not considered. For example, we did not include analysis of cluster-level proportions using linear models with inverse variance weights as, in our experience, it is less frequently used in practice [3]. There are also other choices for standard errors. For example, we did not include GEE with non-robust standard errors as these are widely accepted to be inappropriate. We also did not consider some other variations of the robust sandwich estimator. Of note, the Stata default does not have ‘robust’ as the default option for GEEs. Likewise, given that IEEs do not inherently account for clustering, unless robust standard errors are used, we did not include the IEE approach without robust standard errors. We also included a fairly arbitrary adjustment for clusters with zero events (adding one event to each cluster with zero events), but a wider evaluation of the different approaches might be warranted. Under the cluster-level approaches, we plan to include robust variances when weighting by cluster size but not in the unweighted approach. One argument for using robust standard errors for the unweighted approach is due to different cluster sizes: the different summaries will have different variances (heteroskedasticity). Yet, the unweighted approaches have all worked well with model-based standard errors, even with variable cluster size [30].

## Summary

It is known that different approaches to estimating treatment effects in cluster trials target different estimands, and so the resulting estimate can vary depending on the choice of approach. This is particularly, but not exclusively, the case when estimating odds ratios. In this manuscript, we have described the protocol for a planned re-analysis of data from a large number of cluster randomised trials. This will allow us to examine empirically whether and how odds ratios estimated using different approaches vary in cluster randomised trials.

We cannot aim to make recommendations for given trials. For example, it is likely that we will identify in settings where the ICC is very low that non-allowance for clustering might not underestimate the standard errors [31]. However, in practice, for any given trial, the ICC will not be known with any certainty at the planning stage, and to avoid undue emphasis on model selection, it would not be possible to identify in advance if the ICC were sufficiently low. We suspect that many of our findings will have similar caveats. However, it would be useful, for example, to be able to conclude that large differences are infrequent and mostly only occur when, for example, when ICCs are very large or the variation in cluster sizes is very large. Nonetheless, even if there are no differences between these estimated values, researchers still need to consider how their estimates are going to be interpreted. But of course, the dangers of mis-interpreting are lessened when differences are small.

Thus, we anticipate this work will provide empirical support for known theoretical relationships. This might then be useful in providing guidance to researchers when they need to very carefully consider the question of the target estimand (which in cluster trials is complex to understand) and associated model choice. Moreover, whilst simulation studies and theoretical work can shed light on where differences are anticipated, both of these approaches often, by necessity, make assumptions either about the data generation process or rely on asymptotic or large sample theory. We thus anticipate that this re-analysis and demonstration of empirical findings will complement future and current theoretical work.

## Supplementary Information

Supplementary Material 1. Email to authors of published CRTs to request their data.

Supplementary Material 2: Supplementary Table 1. List of relevant websites known to the authors. Supplementary Table 2. Dummy table for characteristics of the included CRTs. Supplementary Table 3. Approaches not used and justification.

Supplementary Material 3: Supplementary Fig. S1. Confirm that approaches that do not allow for clustering have smaller SEs than other approaches. Supplementary Fig. S2. Determine if models with cluster-robust standard errors have typically larger standard errors than the other approaches.

## Abbreviations

CL: Cluster
CONSORT: Consolidated Standards of Reporting Trials
CRTs: Cluster randomised controlled trials
CV: Coefficient of variation
GEE: Generalised estimating equations
GLM: Generalised linear models
GLMM: Generalised linear mixed models
HC1: Huber-White sandwich estimator
IQR: Inter quartile range
ICC: Intra-cluster correlation coefficient
IEE: Independence estimating equations
LM: Linear model
LMICs: Lower-middle-income countries
OR: Odds ratio
RCTs: Randomised controlled trials
SAPs: Statistical analysis plans
SE: Standard error
