# Supplementary material for: Re-analysis of data from cluster randomised trials to explore the impact of model choice on estimates of odds ratios: study protocol
Source: Trials. 2024 Dec 18;25:818. doi: 10.1186/s13063-024-08653-1 (PMC11653799; doi:10.1186/s13063-024-08653-1)
Supplement: Supplementary file 2 — Supplementary Material 2: Supplementary Table 1. List of relevant websites known to the authors. Supplementary Table 2. Dummy table for characteristics of the included CRTs. Supplementary Table 3. Approaches not used and justification. [file 13063_2024_8653_MOESM2_ESM.docx]

**Supplementary Table 1:**

| **Resource** | **Website** |
| --- | --- |
| NIH National Heart, Lung and blood institute | https://biolincc.nhlbi.nih.gov/studies/ |
| National Institute of Mental Health Data Archive | https://nda.nih.gov/ |
| ClinicalStudyDataRequest.com | https://www.clinicalstudydatarequest.com/Default.aspx |
| DRYAD (open data publishing platform) | https://datadryad.org/stash |
| LSHTM Data Compass | https://datacompass.lshtm.ac.uk/ |
| Harvard Dataverse | https://dataverse.harvard.edu/ |
| B2FIND | https://b2find.dkrz.de/dataset/ |

**Supplementary Table 2: Dummy table for characteristics of the included CRTs**

| **Characteristic** | **LMIC Sample** | **Publicly Available Datasets** | **Datasets known to study team** |
| --- | --- | --- | --- |
| Number studies (n) |  |  |  |
| Number outcomes (n) |  |  |  |
| Average outcome per study (median, IQR) |  |  |  |
|  |  |  |  |
| Total sample size (median, IQR) |  |  |  |
| Number clusters (median, IQR) |  |  |  |
| Cluster size (median, IQR) |  |  |  |
| Coefficient of variation of cluster sizes (median, IQR) |  |  |  |
|  |  |  |  |
| ICC (median, IQR) |  |  |  |
| Control arm proportion (median, IQR) |  |  |  |
| Coefficient of variation of control arm proportions (median, IQR) |  |  |  |
| Correlation between ICC and control arm proportion (median, IQR) |  |  |  |
|  |  |  |  |
| Average point estimate for log-OR (median, IQR)* |  |  |  |
| Average SE for log-OR (median, IQR)* |  |  |  |

ICC: Intra-cluster Correlation Coefficient (reported on the proportions scale and estimated using a linear mixed model); IQR: Inter Quartile Range; OR: Odds Ratio; SE: Standard Error; *Obtained from a GLMM.

**Supplementary Table 3: Approaches not used and justification**

| **Approach** | **Standard Error** | **Target** | **Stata code** | **Justification for exclusion** |
| --- | --- | --- | --- | --- |
| GLM (logit link) with cluster-robust SEs | Clustered-sandwich estimator |  | glm outcome treat, family(binomial) link(logit) vce(cluster cluster) iterate(1000) | Identical to IEE robust, which is included |
| GLM (logit link) with robust SEs | Huber/White/Sandwich estimator |  | glm outcome treat, family(binomial) link(logit) vce(robust) iterate(1000) | This robust standard error corrects for heteroskedasticity and not clustering (so SEs are likely too small) |
| GLMM size weighted |  |  | xtgee outcome treat [pw=1/cluster_size], family(binomial) link(logit) corr(exch) robust iterate(1000) | This approach is rarely used in practice, and in some settings (GEE), using inverse-variance weighting has been found to produce bias (33) |
| IEE (logit link) with non-robust SEs | Naïve |  | xtgee outcome treat, family(binomial) link(logit) corr(independent) iterate(1000) | IEEs do not inherently account for clustering, unless robust standard errors are used (so SEs will be too small). |
| IEE (logit link) with robust standard errors and nmp correction | Robust with *nmp* correction |  | xtgee outcome treat, family(binomial) link(logit) corr(independent) vce(robust) nmp iterate(1000) | Some differences between this SE and the robust, but differences minimal. |
| GEE (logit link) with robust standard errors and nmp correction | Robust with *nmp* correction |  | xtgee outcome treat, family(binomial) link(logit) corr(exchangeable) vce(robust) nmp iterate(1000) | Some differences between this SE and the robust, but differences minimal. |
| Generalised linear mixed model with logit link fitted to binomial data of count of outcomes and total cluster size |  |  | meglm outcome_total treat\|\| cluster:, family(binomial cluster_total) link(logit) | This is grouped binomial data and is identical to the mixed model.  This code can be useful for when only cluster-level data are available, and the object is to target the results from a mixed model. |
| CL with variance weights |  |  | glm logit_prop treat [pweight = var_wt], family(gaussian) link(identity)  where var_wt is [m_i/(1+(m_i-1)*ICC]  where m_i is the size of the cluster and ICC is an estimate of the ICC | This is a cluster-level analysis using ordinary least squares, where the cluster-level proportions are transformed (e.g. to logit proportions), and the logit of the cluster-level proportions is analysed using a linear model with weights that are a function of the ICC. This approach has been found to give different estimates of effects compared to other cluster-level approaches (8, 29, 33) in some settings but identified to work reasonably well in others (30). |
