# Supplementary material for: Re-analysis of data from cluster randomised trials to explore the impact of model choice on estimates of odds ratios: study protocol
Source: Trials. 2024 Dec 18;25:818. doi: 10.1186/s13063-024-08653-1 (PMC11653799; doi:10.1186/s13063-024-08653-1)

**Supplementary Figures**

The figures have been truncated, and outliers have been removed. All results are presented on the log-odds ratio scale.

GLMM: Generalised Linear Mixed Model; GEE: Generalised Estimating Equations (with exchangeable working correlation); IEE: Independent Estimating Equations; CL: Cluster-Level approach (using linear regression of logit of cluster level proportions); wtd: weighted by cluster size; Naïve: generalised linear model (no allowance for clustering); ICC: Intra-cluster Correlation Coefficient; SE: Standard Error; CV: Coefficient of Variation (of cluster sizes).

For full details of approaches, see Table 1. Plots displayed by ICC categories <=0.001; 0.001 to 0.05; 0.05 to 0.1; >=0.1.

**Figure S1 Confirm that approaches that do not allow for clustering have smaller SEs than other approaches**

[
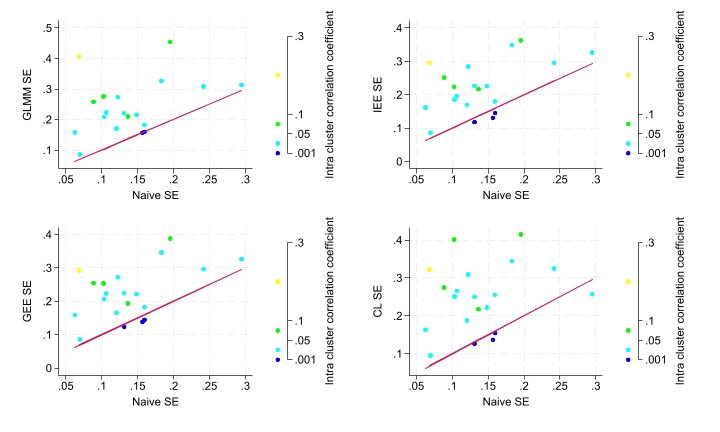
](https://bham-my.sharepoint.com/personal/k_hemming_bham_ac_uk/Documents/Reanalysis%20of%20CRTs/Data%20Analysis/Figures%20Saved%20April%2024/Figure%20S1.jpg)

**Figure S2: Determine if models with cluster-robust standard errors have typically larger standard errors than the other approaches.**


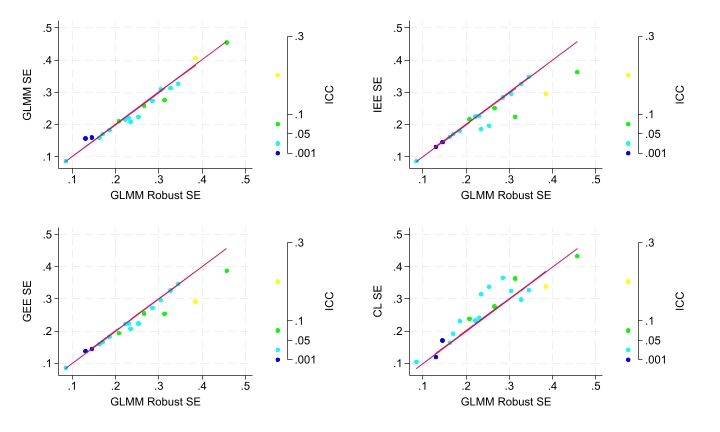

Supplement: Supplementary file 3 — Supplementary Material 3: Supplementary Fig. S1. Confirm that approaches that do not allow for clustering have smaller SEs than other approaches. Supplementary Fig. S2. Determine if models with cluster-robust standard errors have typically larger standard errors than the other approaches. [file 13063_2024_8653_MOESM3_ESM.docx]
