# Supplementary material for: Re-analysis of data from cluster randomised trials to explore the impact of model choice on estimates of odds ratios: study protocol
Source: Trials. 2024 Dec 18;25:818. doi: 10.1186/s13063-024-08653-1 (PMC11653799; doi:10.1186/s13063-024-08653-1)
Supplement: Supplementary file 1 — Supplementary Material 1. Email to authors of published CRTs to request their data. [file 13063_2024_8653_MOESM1_ESM.docx]

**Supplementary Material 1: Email to authors of published CRTs to request their data**

**Subject line:** Your cluster randomized trial

Dear Dr. ________,

We are emailing regarding your cluster randomized trial: [“**Trial Title**”] published in [journal name] in [year]. This trial was identified as part of a scoping exercise to identify published cluster trials. Our research team is working on a methodological project that aims to use real data from a large sample of published cluster randomized trials.

Our primary objective is to empirically explore different methodological approaches for analyzing data from cluster trials. Our aim is to identify if, in practice, model choice can have important consequences for estimating treatment effects. We intend to analyze a large number of datasets (for both primary and secondary binary outcomes) to determine if this is the case.

We are writing to kindly request a de-identified copy of your dataset for our project. We only need a minimal dataset that includes the following variables:

1. Cluster indicator
2. Treatment arm indicator (intervention or control)
3. Any key primary or secondary binary outcome variables
4. Any key primary or secondary continuous outcome variables
5. Any cluster-level variables used in any restricted randomization
6. Any individual or cluster-level variables used in a covariate-adjusted analysis

Please note that our study has been reviewed by the University of Birmingham (UoB) Research Ethics Team. We assure you that all data will be housed on the UoB secure server and will only be available for our research team to use in this project. We will not use the data for general meta-analyses or other large-scale attempts to reinterpret the trials. The results from our analyses will be shared with trial authors upon request. We have attached a copy of our UoB-approved study protocol, which provides more details. We genuinely appreciate your consideration and assistance in this matter, as having your dataset would greatly enhance the quality and comprehensiveness of our project.

If you have any questions or require additional information, please do not hesitate to contact us. We look forward to your positive response.

Sincerely,

Jacqueline Thompson, University of Birmingham, on behalf of the research team:

Professor Karla Hemming, University of Birmingham, UK

Professor Monica Taljaard, University of Ottawa, Canada

Dr Jennifer Thompson, London School of Hygiene & Tropical Medicine, UK

Dr Brennan Kahan, University College London, UK

Professor Andrew Copas, University College London, UK

Dr Sam Watson, University of Birmingham, UK
Dr Jess Kasza, Monash University, UK
